# Effects of electroacupuncture therapy on intractable facial paralysis: A systematic review and meta-analysis

**DOI:** 10.1371/journal.pone.0288606

**Published:** 2023-07-13

**Authors:** Yihao Zhou, Xu Dong, Yating Xing, Ruoyu Wang, Siyu Yang, Yixiao Han, Dongyan Wang

**Affiliations:** 1 Heilongjiang University of Chinese Medicine, Harbin, China; 2 The Second Affiliated Hospital, Heilongjiang University of Chinese Medicine, Harbin, China; University of Catania, ITALY

## Abstract

**Objective:**

This systematic review and meta-analysis aimed to assessment effects of electroacupuncture (EA) therapy on intractable facial paralysis.

**Methods:**

The articles of EA treatment for intractable facial paralysis were retrieved from seven databases, the publication period was from its inception to November 30, 2022. Primary measure was the total effective rate, and other measures included the cure rate, Portmann scores, House-Brackmann scores, Sunnybrook scores and adverse events. The effect size of meta-analysis was expressed using relative risk (RR) or standardized mean difference (SMD) with 95% confidence interval (CI).

**Results:**

A total of 18 studies with 1,119 participants were included, all of them had various aspects of bias risk. Meta-analysis results revealed that EA ways improved total effective rate more effectively compared with non-EA counterparts (RR 1.23, 95% CI 1.17–1.31, *I*^*2*^ = 0%, 18 studies, 1119 participants), and improved cure rate more significantly than non-EA groups (RR 2.04, 95% CI 1.70–2.44, *I*^*2*^ = 0%, 18 studies, 1119 participants). None of studies reported adverse events.

**Conclusion:**

EA therapy is more beneficial for patients with intractable facial paralysis than non-EA, but we lack sufficient evidence to evaluate its safety and follow-up effect. Therefore, more clinical trials with high quality methodologies are needed to further verify long-term effects of EA for IFP and improve the level of evidence.

**Trial registration:**

**Registration number:** CRD42021278541.

## Introduction

Facial paralysis is a peripheral neuropathy resulted from malfunction of facial nerves, patients will have facial asymmetry symptoms ranging from a few days to a few months [1]. Unfortunately, nearly a third of patients still struggle to return to normal after a period of treatment [2]. Patients with facial paralysis in the course of more than two months and the facial muscle function has not been significantly recovered, it is considered to be a stubborn disease, known as intractable facial paralysis (IFP) [3]. Long-term facial asymmetry symptoms bring appearance anxiety to patients, which could affect patients’ social interaction seriously and even lead to depression [4]. Therefore, in order to better ameliorate the patient’s facial asymmetry, it is necessary to take appropriate and more effective therapeutic interventions during treatment.

At present, there have multiple salvage therapy for IFP, including glucocorticoid, neurotrophic drug, botulinum toxin, physical rehabilitation, acupuncture and moxibustion and so on [5,6]. Recent years, people began to pay attention to the application of instrumental physical therapies in the recovery process of patients with facial paralysis, laser with low level and electrical stimulation have been shown to effectively improved paralytic facial muscle function [7].

Acupuncture is one of the representative traditional treatments of Chinese medicine, which has a long history. Manual acupuncture (MA) and electroacupuncture (EA) are two commonly therapeutic modes that are frequently used in clinical [8]. EA is a form of manual acupuncture combined with electrical stimulation, which could achieve more curative effect on account of the uniform and continuous stimulus [9]. A published systematic review has shown that EA is beneficial for facial paralysis and resulting in a better prognosis [10]. However, there is insufficient evidence to support whether EA apply equally to patients with IFP, and not enough evidence to evaluate potential harm. In China, when and how EA is most beneficial for patients with facial paralysis has been a controversial issue. Thus, we expect to carry out a systematic review to assessment effects of EA therapy on IFP, and provide conclusive basis for clinical practice.

## Methods

This study protocol has been registered in the PROSPERO, registration number is CRD42021278541. The protocol has been peer reviewed and published in the PLoS ONE (at journals.plos.org). Our study was strictly implemented adhere to the Preferred Reporting Items for Systematic Reviews and Meta-Analyses (PRISMA) reporting guidelines [11]. The PRISMA Checklist is shown in S1 Appendix.

Data synthesis was performed applied the RevMan5.3 software. Dichotomous data were expressed as relative risk (RR) and continuous variables as standardized mean difference (SMD) with 95% confidence interval (CI). All included studies was evaluated bias risk by the Cochrane Handbook. The evidence certainty was assessed by the Grading of Recommendations Assessment, Development and Evaluation (GRADE).

Additionally, we revised the following two points of information in the protocol and implemented the new plan, other methods were executed in accordance with the registered protocol exactly. More details of statistical analysis plan are presented in S2 Appendix.

·We extended the deadline of publication period from March 2022 to November 30, 2022 because our work officially begins after the protocol has been peer-reviewed.
·We added and analyzed two secondary outcome measures, Portmann scores and Sunnybrook scores, based on the characteristics of included studies.

## Results

### Study selection

**Fig 1:**
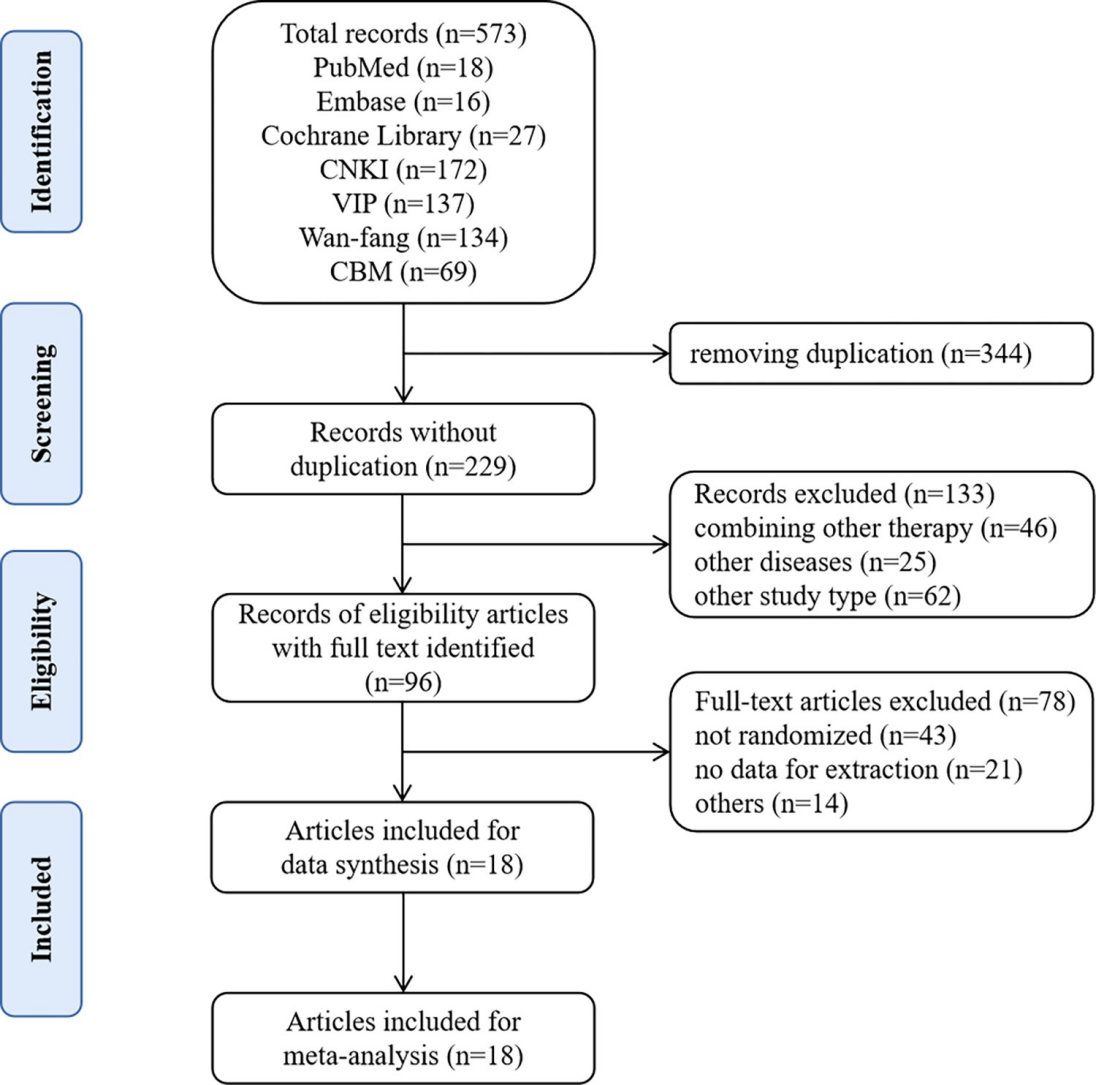
The details of the selection process.

According to the search strategy, 573 records were retrieved from seven databases, 344 duplicate records were identified and removed, 133 irrelevant records were excluded by screening tittle and abstract, further 78 records were excluded after screening full-text articles. Finally, 18 records were included in our study. The details of the study selection are shown in Fig 1.

### Study characteristics

A total of 18 studies with 1,119 participants, included the treatment group was 574, and the control group was 545. Each study have used different interventions in the treatment group, six studies used only EA [12–17], and the remaining twelve studies combined with other interventions, such as acupoint injection (AI) [18–22], hyperbaric oxygen (HO) [23], traditional Chinese medicine drugs (TCMD) [18,24,25], conventional therapy (CT) [26,27], blood-letting therapy (BLT) [28], and ultrashort wave (UW) [29]. The intervention ways of the control group were MA, HO, TCMD or CT. All studies choose the total effective rate and the cure rate as the outcome measures, three studies reported Portmann scores [15,24,28], two studies reported House-Brackmann scores [13,27], and one study reported Sunnybrook scores [12]. However, there was no study reported adverse events. The characteristics of all 18 studies are shown in Table 1.

**Table 1:** The characteristics of included trials.

Study	Country	Sample size (I/C)	Disease course	Intervention		Outcome measures
				I	C	
Cao and Sun, 2019 [12]	China	20/20	≧3 month	EA	MA	Total effective rate, Cure rate, Sunnybrook scores
Shang and Zhao, 2018 [28]	China	25/25	≧3month	EA+BLT	MA	Total effective rate, Cure rate, Portmann scores
Zhang et al., 2017 [13]	China	30/30	≧2month	EA	MA	Total effective rate, Cure rate, H-B scores
Li et al., 2016 [23]	China	39/37	2-8month	EA+HO	HO	Total effective rate, Cure rate
Tan, 2016 [26]	China	20/20	4-10month	EA+CT	CT	Total effective rate, Cure rate
Chen, 2015 [29]	China	20/21	≧2month	EA+UW	MA	Total effective rate, Cure rate
Meng et al., 2015 [24]	China	17/17	≧2month	EA+TCMD	TCMD	Total effective rate, Cure rate, Portmann scores
Lin et al., 2014 [14]	China	34/35	≧3month	EA	MA	Total effective rate, Cure rate
Zhong, 2014 [18]	China	18/18	2-36month	EA+AI+TCMD	CT	Total effective rate, Cure rate
Song, 2014 [15]	China	30/30	3-8month	EA	MA	Total effective rate, Cure rate, Portmann scores
Bao et al., 2010 [19]	China	32/30	2-6month	EA+AI	MA	Total effective rate, Cure rate
Li, 2008 [25]	China	100/89	≧2month	EA+TCMD	TCMD	Total effective rate,Cure rate
Zhu et al., 2004 [27]	China	20/17	≧3month	EA+CT	CT	Total effective rate, Cure rate, H-B scores
Zhang et al., 2004 [21]	China	30/30	≧2month	EA+AI	MA	Total effective rate, Cure rate
Jin et al., 2003 [20]	China	25/25	5-14month	EA+AI	MA	Total effective rate, Cure rate
Yang, 2003 [16]	China	42/32	≧3month	EA	MA	Total effective rate, Cure rate
Chen, 2002 [22]	China	35/30	3-11month	EA+AI	MA	Total effective rate, Cure rate
Tao and Zhang, 2000 [17]	China	37/39	≧2month	EA	MA	Total effective rate, Cure rate

I: Intervention group; C: Control group. EA: Electric acupuncture; MA: Manual acupuncture; HO: Hyperbaric oxygen; CT: Conventional therapy; BLT: Blood-letting therapy; TCMD: Traditional Chinese medicine drugs; AI: Acupoint injection; UM: Ultrashort wave.

The EA parameters were different in all 18 trials. In terms of waveform, twelve studies selected dilatational wave [13–17,19,22,24–26,28,29], one study selected discontinuous wave [12], one study selected sharp wave [21], and four of the studies did not specifically describe waveform [18,20,23,27]. In terms of stimulation time, fourteen studies used 30 minute [12–14,16,17,19,21,22,24–29], other four studies selected 15min [18], 40min [15] or not mentioned [20,23]. About the stimulation strength, twelve studies were described as a patient tolerance [12–17,21,22,26–29], and the remaining studies were not mentioned. The choice of EA acupoints was different in all studies. Most acupoints were located on one side of the paralyzed face, Cuanzhu (BL2) and Yangbai (GB14) were the two most frequently used acupoints, the number of treatment sessions varied from 10 to 60 times. The EA parameters characteristics are shown in Table 2.

**Table 2:** The EA parameters characteristics.

Study	Waveform	Strength	Time (minute)	EA acupoints	Sessions
Cao and Sun, 2019 [12]	Discontinuous wave	Tolerance	30	RN24, ST4, BL2, GB14.	30
Shang and Zhao, 2018 [28]	Dilatational wave	Tolerance	30	ST4, ST7, EX-HN5, GB14.	15
Zhang et al., 2017 [13]	Dilatational wave	Tolerance	30	GB14, BL2, ST2, SJ17, ST4, ST6.	28
Li et al., 2016 [23]	—	—	—	SJ17, ST2, BL2, GB14, LI20, ST4, ST6, Qianzheng.	50
Tan, 2016 [26]	Dilatational wave	Tolerance	30	LI19, SI18, BL2, GB14.	30
Chen, 2015 [29]	Dilatational wave	Tolerance	30	ST4, RN24, LI20, ST7, GB14, BL2.	30
Meng et al., 2015 [24]	Dilatational wave	—	30	GB14, EX-HN4, SJ23, ST2, LI20, SI18, Qianzheng, DU26, ST6, ST4, RN24, ST7.	28
Lin et al., 2014 [14]	Dilatational wave	Tolerance	30	GB14, EX-HN4, ST4, ST7	30
Zhong, 2014 [18]	—	—	15	GB14, ST4, SJ17, ST6.	10
Song, 2014 [15]	Dilatational wave	Tolerance	40	GB14, BL2, ST4, ST6, DU26, LI19, RN24, ST7, LI20.	24
Bao et al., 2010 [19]	Dilatational wave	—	30	SJ17, ST4, GB14, ST2, GB12, ST6, SI18, ST8, GB1, Qianzheng.	30
Li, 2008 [25]	Dilatational wave	—	30	ST4, ST6, GB14, BL2.	50
Zhu et al., 2004 [27]	—	Tolerance	30	BL2, ST1, ST4, Qianzheng.	39
Zhang et al., 2004 [21]	Sharp wave	Tolerance	30	SJ17, GB12, EX-HN5, ST6, ST4, ST7.	60
Jin et al., 2003 [20]	—	—	—	DU20, MS6.	30
Yang, 2003 [16]	Dilatational wave	Tolerance	30	ST4, LI20, GB14, BL2, ST7, RN24.	10
Chen, 2002 [22]	Dilatational wave	Tolerance	30	GB14, EX-HN5, ST1, LI20, ST4, ST6.	30
Tao and Zhang, 2000 [17]	Dilatational wave	Tolerance	30	GB14, BL2, ST2, ST4, SI18, RN24.	20

### Study design and risk of bias

**Fig 2:**
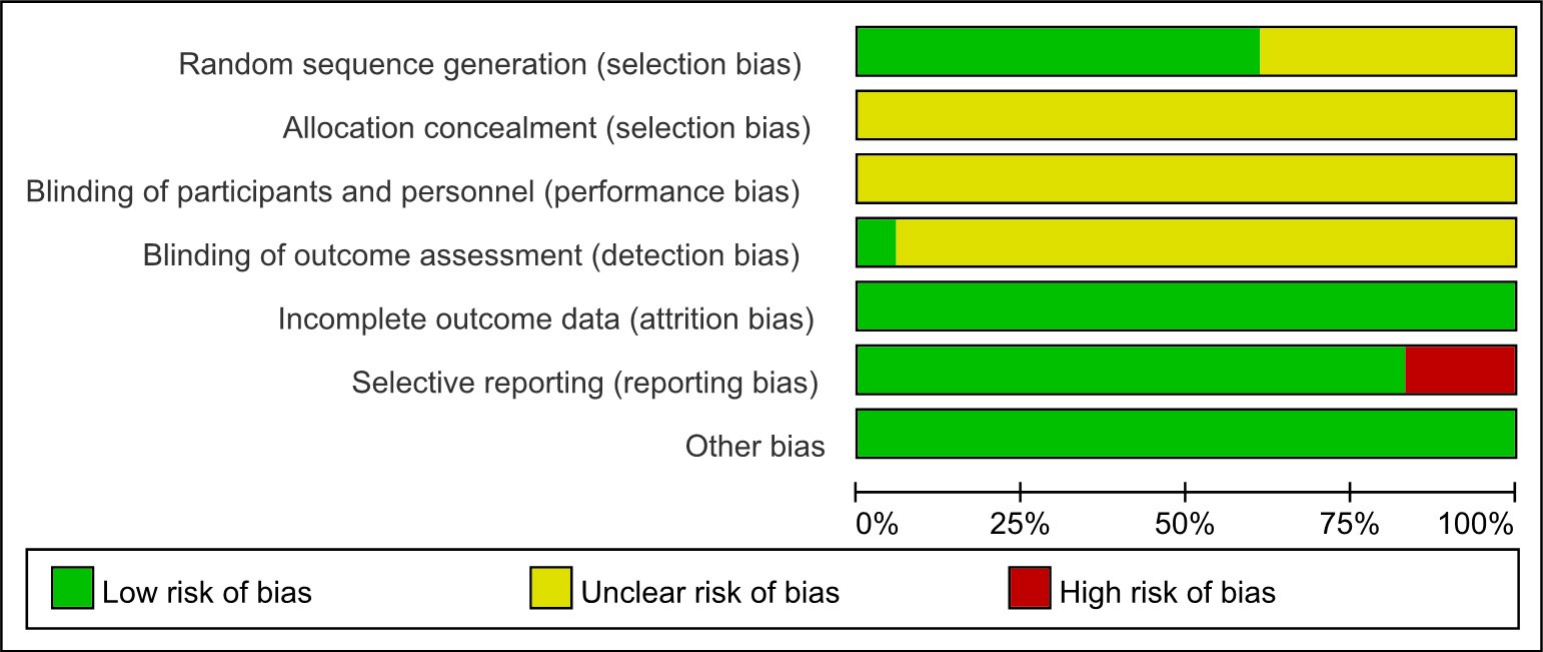
Risk of bias graph.

All studies had various aspects of bias risk because of methodological deficiencies (Fig 2). Four studies used randomized digital tables [13–15,25], five studies randomized in order of visited [12,16,19,21,28], two studies randomized according to lots [18,24], and seven studies were evaluated as unclear risk due to absence details of random sequence generation. All studies were considered unclear risk as lack of allocation concealment process. Blinding was difficult as the particularity of acupuncture, and all studies absence details on how to blind patients and physicians. In terms of detection bias, only one study [24] adopted blinding of outcome assessment and judged as low risk. Even so, in the opinion of our review team after discussion and evaluation, randomization and blinding appeared to be successful due to there was almost none discrepancy in the baseline. No studies reported cases of dropouts or eliminations, thus they were judged as low risk in attrition bias. Three studies [15,26,29] adopted House-Brackmann scores but didn’t report this result and therefore the reporting bias was high risk. No other bias was found in any of the studies.

### Primary outcomes

#### The total effective rate

**Fig 3:**
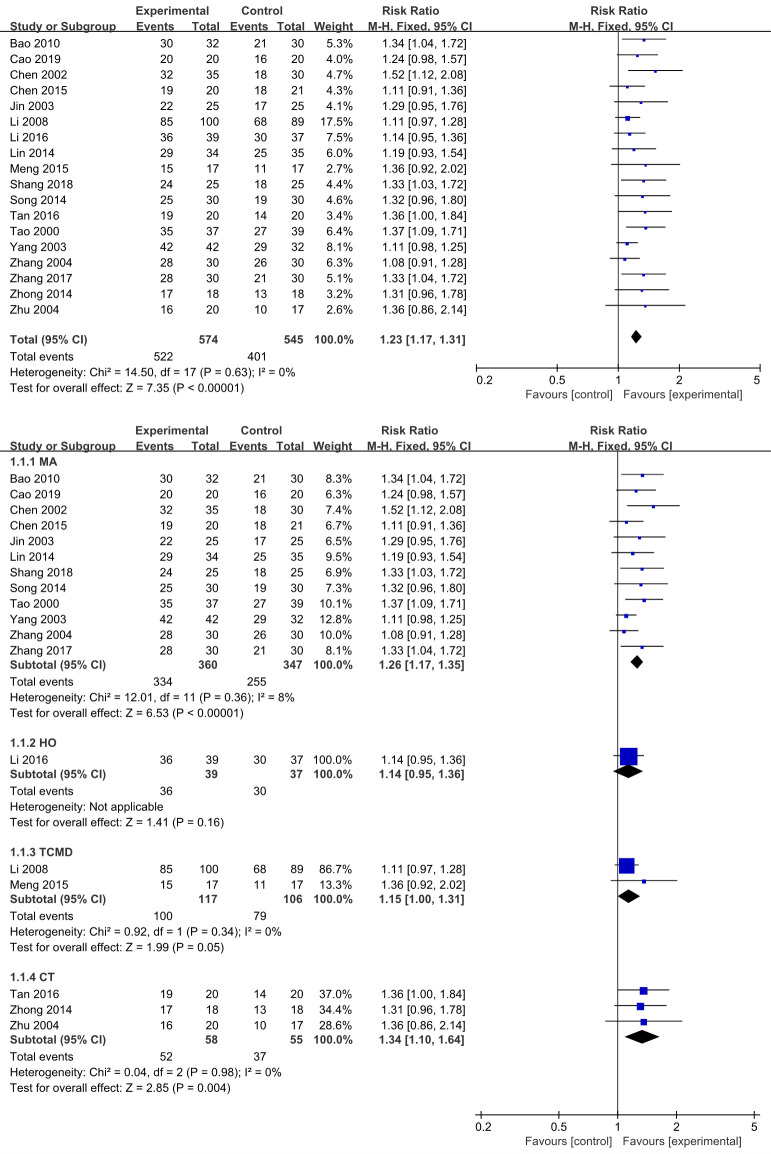
A. Forest plots of the total effective rate in the EA vs non-EA. B. Forest plots of the total effective rate (subgroup analysis).

**Fig 4:**
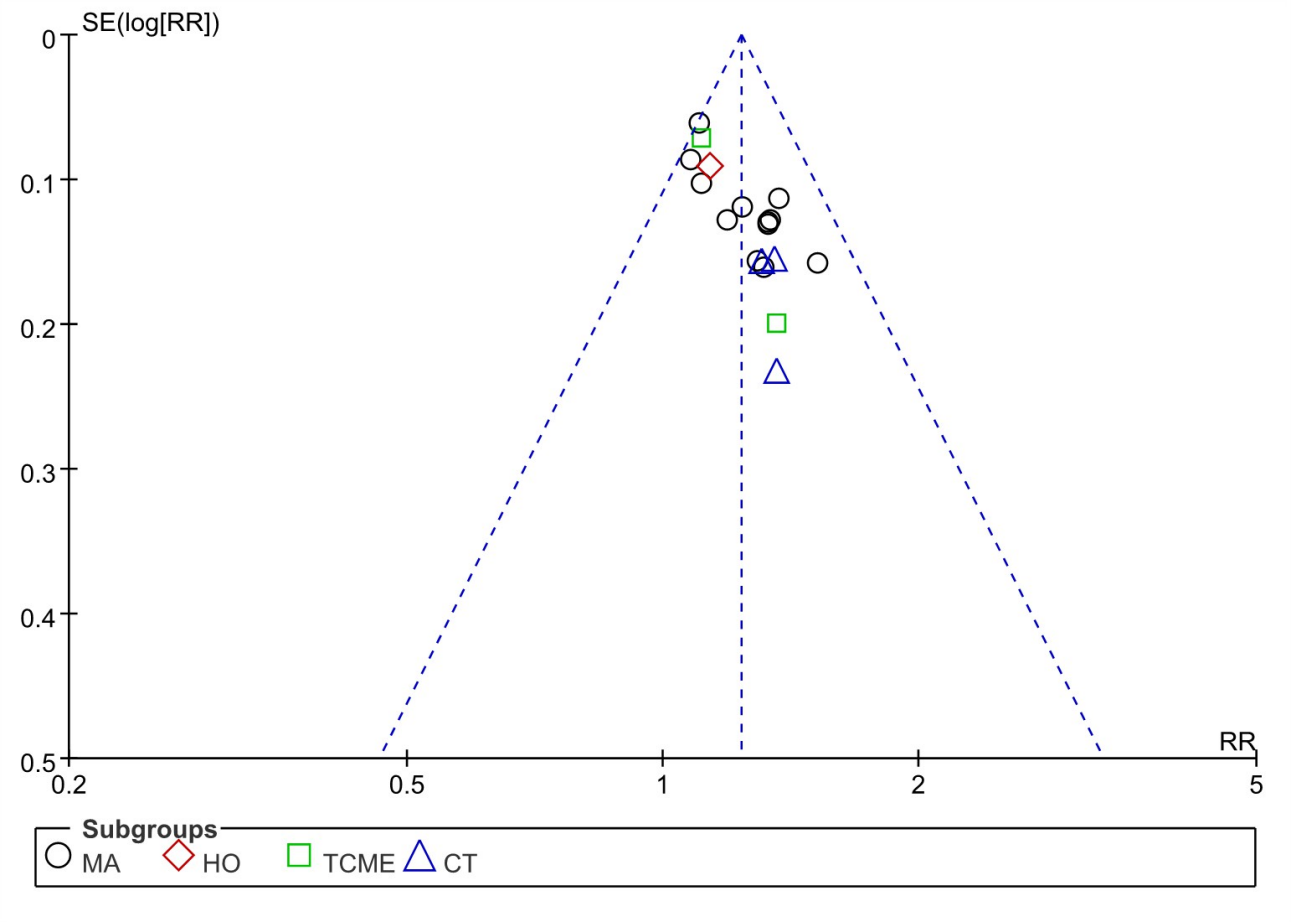
Funnel plot of the total effective rate.

All studies reported this result, we performed a meta-analysis of these data from 18 studies. The results revealed that EA ways improved total effective rate more effectively compared with non-EA counterparts (RR 1.23, 95% CI 1.17–1.31, *I*^*2*^ = 0%, 18 studies, 1119 participants) (Fig 3A). Considering there have a variety of intervention ways to treatment for IFP in clinical, we further conducted a subgroup analysis according to type of treatment methods in the control group (Fig 3B). The results revealed that EA was significantly beneficial than MA (RR 1.26, 95% CI 1.17–1.35, *I*^*2*^ = 8%, 12 studies, 707 participants) and CT (RR 1.34, 95% CI 1.10–1.64, *I*^*2*^ = 0%, 3 studies, 113 participants), but EA was little or no differences versus HO (RR 1.14, 95% CI 0.95–1.36, 1 study, 76 participants) and TCMD (RR 1.15, 95% CI 1.00–1.31, *I*^*2*^ = 0%, 2 studies, 223 participants). The heterogeneity regarded as low because of *I*^*2*^ < 50%, it suggested the results were reliable, but the funnel plot showed a slight asymmetry (Fig 4).

### Secondary outcomes

#### The cure rate

**Fig 5:**
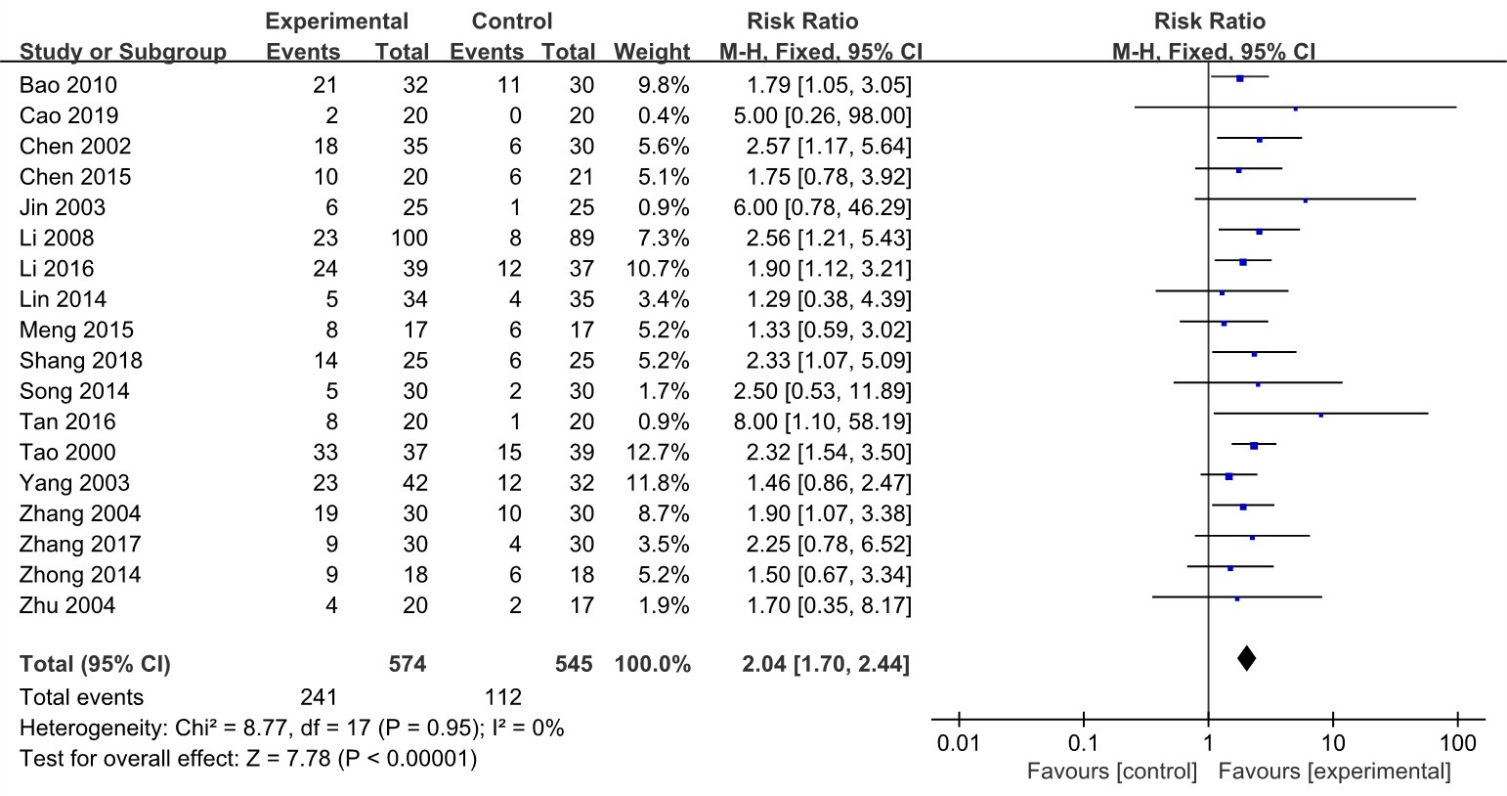
Forest plots of the cure rate in the EA vs non-EA.

All studies reported the cure rate, and the results indicated that EA ways improved cure rate more significantly, it was 2.04 times more effective than non-EA groups (RR 2.04, 95% CI 1.70–2.44, *I*^*2*^ = 0%, 18 studies, 1119 participants) (Fig 5). Meanwhile, the results of heterogeneity revealed that this result was reliable.

#### Portmann scores

**Fig 6:**
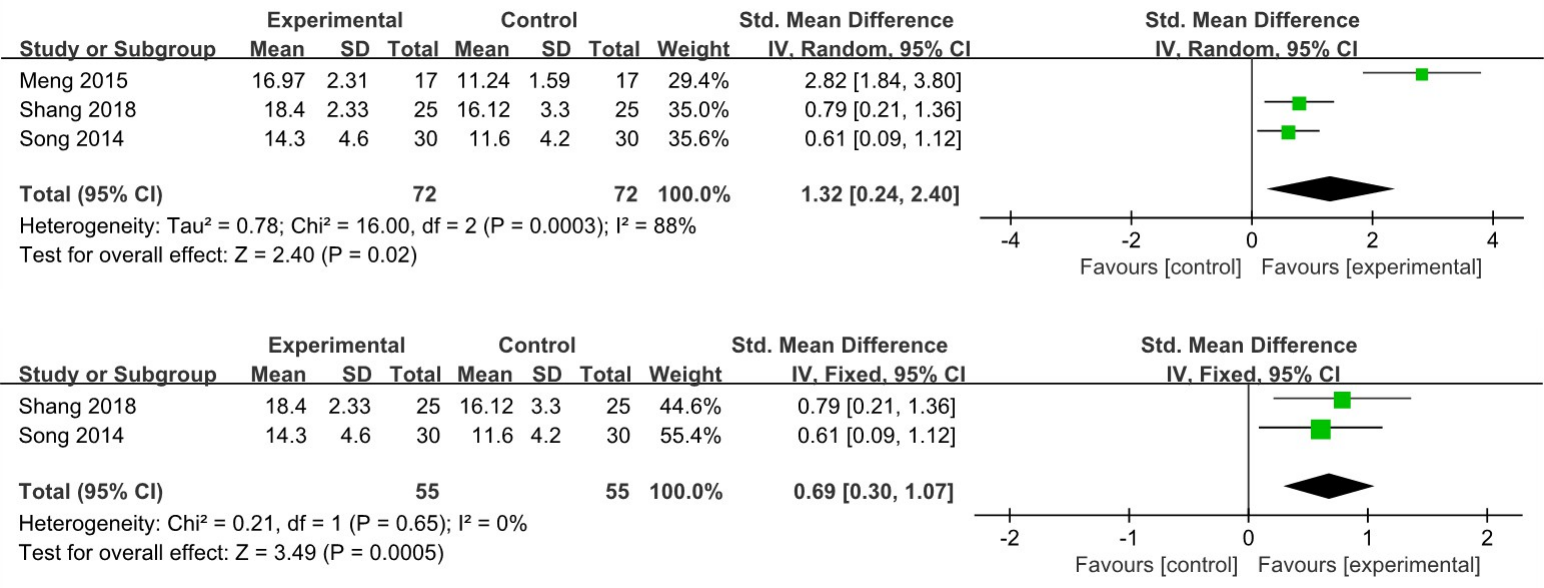
A. Forest plots of Portmann scores in the EA vs non-EA. B. Forest plots of Portmann scores in the EA vs non-EA after removed one study.

Three studies [15,24,28] reported Portmann scores, results showed that EA could improvement the symptom scores (SMD 1.32, 95% CI 0.24–2.40, *I*^*2*^ = 88%, 3 studies, 144 participants) (Fig 6A). Sensitivity analysis of this result discovered the heterogeneity between the remaining studies was reduced and the result was reliable when Meng et al. 2015 [24] was excluded (SMD 0.69, 95% CI 0.30–1.07, *I*^*2*^ = 0%, 2 studies, 110 participants) (Fig 6B).

#### Other outcome measures

Two studies [13,27] reported House-Brackmann scores, and sensitivity analysis showed the heterogeneity was significant (*I*^*2*^ = 97%, *P*<0.1). Meanwhile, only one study [12] reported Sunnybrook scores. We have don’t perform meta-analysis in House-Brackmann and Sunnybrook scores cause the heterogeneity between studies was significant and difficulty to incorporate the analysis.

#### Adverse events

None of studies reported adverse events, it was not conducive to our evaluation of the safety of EA for IFP, and there may be a potential risk of reporting bias.

### Certainty of evidence

**Fig 7:**
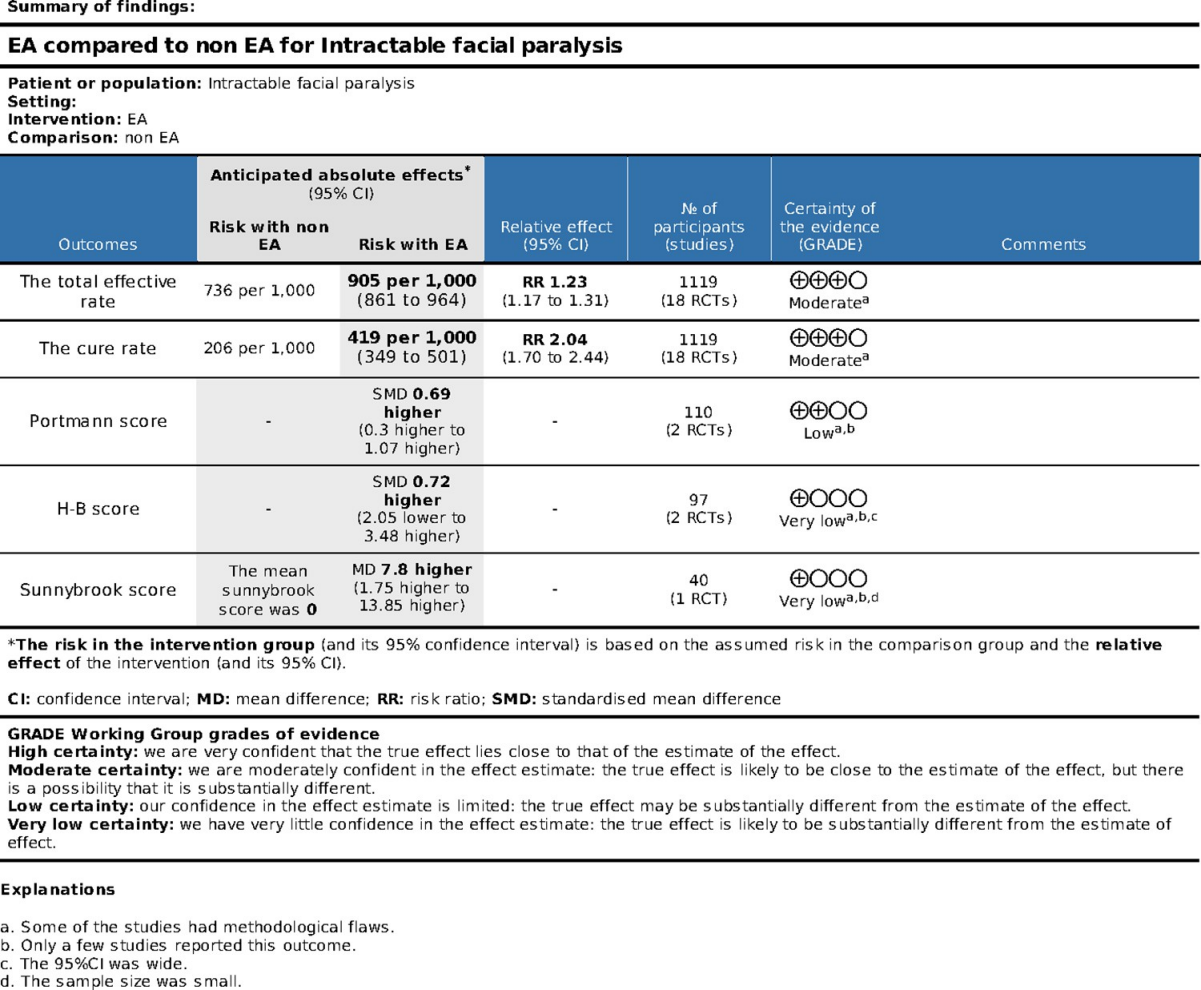
Level of evidence (GRADE).

The evidence certainty was assessed by GRADEpro GDT, its which indicated the evidence level of all measures ranged from moderate to very low, more details were available in Fig 7.

## Discussion

### Main findings

This systematic review investigated the effects of EA therapy on IFP relative to non-EA. After analyzing 18 studies covering 1,119 participants, the results indicated that EA ways can improved total effective rate significantly, especially when compared with MA and CT group, but there was little or no differences in clinical effect versus HO and TCMD group. Similar findings in the outcome measures of cure rate. There was no study reported adverse events, thus we lack sufficient evidence to evaluate its safety.

### Interpretation

To date, modern medicine has no unified understanding of the etiology of IFP. Many researchers believe that its pathogenesis is closely related to several reasons. The first reason is the degree of facial nerve injury. Severe injury will not only prolong the treatment period, but also reduce the possibility of cure [30]. Secondly, early and middle course of the disease adopt inappropriate treatment methods is a another important reason, its which fails to effectively improve the pathological state of facial nerve ischemia and hypoxia, cause it difficult for facial muscle function to restore normal, and may even cause more severe sequelae [31]. Finally, the patient’s immunity, environmental climate and other factors may also affect process of recovery.

Acupuncture is a widely used complementary and alternative treatment method in the world, which has been proven to show significant effects in peripheral neuropathy. The World Health Organization strongly recommends the use of acupuncture in the treatment of facial paralysis [32]. Modern science has provided biological evidence for the effects of acupuncture, revealing that acupuncture treatments, including electrical acupuncture, can ultimately regulate physiological function by activating peripheral neural pathways [33]. EA could through acupuncture combined with current stimulation, rhythmically relax local muscles, to better regulate tissue cell function, strengthen blood-lymphatic circulation as well as ion movement, accelerate waste metabolism, increase tissue nutrition, eliminate inflammatory edema, and thus promoting damaged nerve and muscle repair and regeneration [34,35]. In addition, it could also promoting axons growth, regulating vasoconstriction or hemodynamics, and eventually restore the function of the injured facial nerves [36]. These pathways and mechanisms may be the biological basis for the more effective EA on IFP.

### Publication bias

The funnel plot of the total effective rate showed a slight asymmetry, suggesting a potential risk of publication bias or other bias in inclusion trials. Generally, the asymmetric resulted may be due to positive research results were easier to be published and thus exaggerating the effect size. Although we attempted to find some unpublished trials with negative results, we found none. Besides, all trials were from the Chinese database, language and regional could also cause bias. Finally, clinical heterogeneity such as differences in intervention methods between trials may be another potential factor for asymmetry that cannot be ignored.

### Quality of evidence

The evidence quality of the total effective rate and the cure rate were moderate, and another outcomes level ranged from low to very low. After discussion, our review team believes that the reasons leading to the decline of evidence level in this study mainly include: (a) Some of the studies had methodological flaws, such as not providing details about allocation concealment, blinding or random method; (b) Only a few studies reported partial outcome, resulting in a low level of evidence for this project; (c) The 95%CI was wide; (d) The sample size was small. Therefore, although this study results showed that EA is beneficial for patient with IFP, we still need to use randomized controlled trials of high-quality methodologies to enhance the grades of evidence even further.

### Strengths and limitations

This study systematically reviews the published literature to explore the effects of EA therapy on IFP, and to comprehensively evaluate its by using multiple outcome measures, which can provide conclusive basis for clinical practice. On the other hand, the methodology of this study have been registered in advance and peer-reviewed, which reduces the risk of bias and improves the credibility of this study.

However, there are unavoidable limitations in this systematic review. Most of trials had risk of bias in randomization and blinding due to lack of details. The possible selective reporting is another important flaw that should not be overlooked. Meanwhile, all the researchers and participants were from China, and these studies were reported in Chinese, we lacked data support from other countries and multiple languages. Last but not least, meta-analysis frequently necessitates the synthesis of the findings from multiple trials, which raises the potential for random mistakes. Random mistake may have contributed to certain "positive" meta-analysis results [37]. Meta-analysis can benefit from trial sequence analysis to investigate the robustness of meta-analysis results and the need for further research, which deserves further attention in future studies.

### Conclusion

This systematic review suggests that EA therapy is more beneficial than non-EA for patient with IFP. It also suggests that acupuncture treatment mode should be selected according to different disease periods, and EA should be used in advance in the middle and late stage of facial paralysis, which may be helpful for patients. However, it’s worth noting that our findings ought to be interpreted prudently because of the generally poor-quality methodologies of all included trials. Moreover, we didn’t find enough evidence to assess the safety, and also lack of follow-up data. Therefore, more clinical trials with high quality methodologies are needed to further verify long-term effects of EA for IFP and improve the level of evidence.

## Supporting information

S1 Appendix The PRISMA checklist. (DOCX)

S2 Appendix Statistical analysis plan. (PDF)

S1 File (XLSX)
