# Supplementary material for: Effects of electroacupuncture therapy on intractable facial paralysis: A systematic review and meta-analysis
Source: PLoS One. 2023 Jul 13;18(7):e0288606. doi: 10.1371/journal.pone.0288606 (PMC10343152; doi:10.1371/journal.pone.0288606)
Supplement: S2 Appendix — (PDF) [file pone.0288606.s002.pdf]

PLOS ONE

REGISTERED REPORT PROTOCOL

Efficacy and safety of electric acupuncture in treatment of intractable facial paralysis: A protocol for systematic review and meta-analysis

Yihao Zhou[1,2], Gan Huang[3], Chunhong Luo[3], Zhilin Huang[3], Anhong Dai[2,3], Xuelian Zhang[3], Meifang Liu[2], Jing Shi[2,3]*

1 Heilongjiang University of Chinese Medicine, Harbin, Heilongjiang, China, **2** Yunnan Provincial Hospital of Traditional Chinese Medicine, Kunming, Yunnan, China, **3** Yunnan University of Traditional Chinese Medicine, Kunming, Yunnan, China

* 2662831291@qq.com

Check for updates

This is a Registered Report and may have an associated publication; please check the article page on the journal site for any related articles.

OPEN ACCESS

Citation: Zhou Y, Huang G, Luo C, Huang Z, Dai A, Zhang X, et al. (2022) Efficacy and safety of electric acupuncture in treatment of intractable facial paralysis: A protocol for systematic review and meta-analysis. PLoS ONE 17(12): e0278509. https://doi.org/10.1371/journal.pone.0278509

Editor: Alessandro de Sire, University of Catanzaro: Universita degli Studi Magna Graecia di Catanzaro, ITALY

Received: July 17, 2021

Accepted: November 18, 2022

Published: December 1, 2022

Copyright: © 2022 Zhou et al. This is an open access article distributed under the terms of the Creative Commons Attribution License, which permits unrestricted use, distribution, and reproduction in any medium, provided the original author and source are credited.

Data Availability Statement: All relevant data from this study will be made available upon study completion.

Funding: This work was supported by Health Science and Technology Program of Yunnan Province (2017NS157), Key Laboratory of Acupuncture and Tuina for Prevention and

Abstract

Background

Facial paralysis is a common clinical disease, it was named intractable facial paralysis when the clinical course more than 2 months. Intractable facial paralysis will produce anxiety and depression, which will seriously affect patients' life and work. Electric acupuncture has been widely used in the treatment of intractable facial paralysis. However, the results of clinical studies on the efficacy and safety have been inconsistent. This study aims to evaluate the efficacy and safety of electric acupuncture for intractable facial paralysis patients by systematic review and meta-analysis, so as to provide clinical decision-making based on evidence-based medicine.

Methods

The following databases will be searched by electronic methods: PubMed, Embase, Cochrane Library, Chinese National Knowledge Infrastructure, VIP Database, Wan-fang Data and Chinese Biomedical Database. All of them will be retrieved from the establishment date of the electronic database to March 2022, all included studies will be evaluated risk of bias by the Cochrane Handbook. The total effective rate will be the primary outcome. The systematic review will be conducted with the use of the RevMan5.3 software in this study.

Results

This study will obtain efficacy and safety of electric acupuncture for the treatment of intractable facial paralysis.

Discussion

This study will provide clinical decision-making based on evidence-based medicine that whether electric acupuncture could be used to treat intractable facial paralysis, and when

Treatment of Encephalopathy in Universities of Yunnan Province (No.2019YGZ04), Technology Innovation Team of Acupuncture Prevention and Treatment of Psychosis in Universities of Yunnan Province (No.2019YGC04). The funders had and will not have a role in study design, data collection and analysis, decision to publish, or preparation of the manuscript.

Competing interests: The authors have declared that no competing interests exist.

Abbreviations: FP, Facial paralysis; IFP, Intractable facial paralysis; MA, Manual acupuncture; EA, Electric acupuncture; RCTs, randomized controlled trials; PRISMA-P, Preferred Reporting Items for Systematic Reviews and Meta-Analyses Protocols; CNKI, Chinese National Knowledge Infrastructure; CBM, Chinese Biomedical Database; RR, Relative risk; SMD, Standardized mean difference; CI, Confidence interval; GRADE, Grading of Recommendations Assessment, Development and Evaluation; WHO, World Health Organization.

and how it might be more effective and safety. It will help standardize electric acupuncture treatment strategies for intractable facial paralysis.

PROSPERO registration number

CRD42021278541.

Introduction

Description of the condition

Facial paralysis (FP) is a common clinical disease caused by damage to the lower motor neurons of the facial nerve, the clinical symptoms mainly include forehead lines and nasolabial sulcus on the affected side into shallowness or disappearance, additional symptoms include incomplete eyelid closure, taste impairment, ear pain, facial numbness and so on [1, 2]. According to reports, the incidence is described as 11.5 to 40.2 per 100000 people a year [3]. Affected patients may experience facial asymmetries that last for weeks to months. One study indicated that only 71% of patients with FP were able to regain normal facial function with treatment, while 29% of patients still had hemifacial weakness [4]. Usually, it was named intractable facial paralysis (IFP) when the clinical course of FP more than 2 months [5]. IFP is one of the stubborn clinical diseases, which has the characteristics of long course and difficult treatment. From the perspective of social psychology, IFP will produce anxiety and depression, which will seriously affect patients' life and work [6]. Therefore, to improve the symptoms and life quality of patients, appropriate treatment interventions are needed in the treatment.

Description and function of intervention

To date, there is a wide variety of salvage therapeutical approaches, including antiviral drugs, corticosteroids, vitamin B12, acupuncture or dry needling, botulinum toxin, and rehabilitation [7, 8]. In the last years, a high interest has been growing regarding the use of instrumental physical therapies, such as lower-level laser therapy and electrical stimulation, in improving facial muscle function in patients with facial palsy [9].

Acupuncture is a characteristic therapy of traditional Chinese medicine. Clinically, there are two commonly used acupuncture interventions methods: manual acupuncture (MA) and electric acupuncture (EA). In China, many studies have claimed that these therapies have exact curative effect for IFP [10, 11]. EA is a treatment that combines MA and electric stimulation, which is more suitable for the treatment of IFP due to the stable and continuous stimulus quantity [12]. Studies have found that EA can regulate the relationship between endothelin secreted by vascular endothelial cells and microcirculation, thereby regulating vasoconstriction or hemodynamics, and ultimately treating IFP [13]. In addition, EA can also speed up the facial nerve's conduction speed, promote the expression of nerve growth factor protein, brain-derived neurotrophic factor protein, BDNF mRNA and its protein in the facial nerve nucleus, thereby enhancing facial nutrition, it also plays a role in nerve regeneration and repair after injury by promoting the growth of facial nerve axons [14].

Why it is important to do this review

Many randomized controlled trials (RCTs) and meta-analyses show that EA treatment has specific benefits for IFP patients [15, 16]. Although the proportion of the application of

acupuncture for IFP is also on the rise [16], there has not been a rigorously designed systematic review and meta-analysis to evaluate the curative effect and potential harm of IFP by EA. Therefore, we hope to conduct a systematic review and meta-analysis for the published literature, to explore the status of EA therapy in IFP treatment and the benefits or harm it may bring to patients. The results of this study could resolve the dispute over the benefits or harm of EA in patients with IFP, and to provide concluding evidence for clinical practice.

Methods

Study registration

This study has been registered on the International Prospective Register of Systematic Reviews in PROSPERO (registration number is CRD42021278541), and will adhere to the guidelines of the Preferred Reporting Items for Systematic Reviews and Meta-Analysis Protocols statement (PRISMA-P) [17]. The PRISMA-P checklist is shown in the S1 Appendix.

Inclusion and exclusion criteria

Type of study. All Clinical RCTs will be included, without restrictions on country but language will be limited on English and Chinese. Reviews, animal experiments, theory discussion, case reports, conference articles, and other non-RCTs study will be excluded.

Type of participants. This study will only include participants with Bell's palsy, the clinical course more than 2 months. Regardless of gender, age, occupation, education, and severity, etc. Patients with central facial paralysis will be excluded.

Type of intervention. EA should be treated in the experimental group and non-EA in the control group. Drug treatment was acceptable in all groups.

Type of outcome measures. (1) *Primary outcome*. The total effective rate of EA to treat IFP will be the primary outcome. The total effective rate = remove invalid subjects/all subjects. (2) *Secondary outcome*. Secondary outcomes mainly include the following aspects: Cure rate, House-Brackmann score, Adverse events incidence. The cure rate = cure subjects/all subjects.

Search strategy. We will search systematically in the following 7 databases: PubMed, Embase, Cochrane Library, Chinese National Knowledge Infrastructure (CNKI), VIP Database, Wan-fang Data, Chinese Biomedical Database (CBM). All of them will be retrieved from the establishment date of the electronic database to March 2022. The retrieval mode used will be a combination of medical subject words and free words, and appropriate retrieval pattern will be conducted in different databases. The search strategy is shown in the S2 Appendix.

Data collection

Study selection. We plan to conduct a systematic review between August 31, 2021 and December 31, 2022. The selection of study, which includes literature screening, data extraction, management and examination, will be conducted by 2 reviewers. They will independently screen the titles, abstracts, and keywords of all retrieved studies and determine which trials meet the inclusion criteria, full texts of all possible relevant studies will be obtained for further evaluation. Any disagreements will be resolved by discussion between the 2 reviewers and the third author. The flow diagram of the study selection process is shown in the S1 Fig.

Assessment of risk of bias in included studies. All included studies will be evaluated risk of bias by the Cochrane Handbook, the judgment of risks will be evaluated and described from the following 6 items, including generation of random sequences, allocation concealment, blinding, incomplete outcome data, selective reporting, and other biases. Risk will be divided into 3 levels: "low risk of bias", "high risk of bias" and "unclear risk of bias".

Dealing with missing data. If trial data is insufficient or missing, the corresponding author will be contacted by email. If missing data is not be available or the author cannot be contacted, these studies will be excluded. We will conduct a limited analysis based on available data and discuss the potential impact of missing data.

Data synthesis

This study will be conducted with the use of the RevMan5.3 software. Dichotomous data were expressed as relative risk (RR) and continuous variables as standardized mean difference (SMD) with 95% confidence interval (CI). We will use I^2 to evaluate statistical heterogeneity between trials. The heterogeneity will be regarded as high ($I^2 > 75\%$), moderate (I^2 between 50% and 75%), and low ($I^2 < 50\%$). The random-effects model will be used for the merger analysis when the heterogeneity is moderate, and the fixed-effects model will be used when the heterogeneity is low. If the I^2 value is higher than 75%, the clinical or methodological heterogeneity will be explored through discussion with the review team. When the meta-analysis is not possible, a narrative analysis will be performed.

Subgroup analysis

If there are a large number of subgroup studies, a subgroup analysis is performed to determine heterogeneity. It may be includes the method of electric acupuncture frequency, the choice of acupoints, the duration and severity of the disease, etc.

Sensitivity analysis

We will conduct a sensitivity analysis to verify the robustness of the preliminary results by a revaluation of risk of bias, methodological quality, missing data or other possible factors. If there is a large difference, sensitivity analysis will be employed careful interpretation.

Publication bias

If more than 10 studies are included in the meta-analysis, we will evaluate the publication bias by EGGER regression test, and the evaluation will be presented in the form of funnel plots.

Grading the quality of evidence

We will evaluate the quality of evidence according to the Grading of Recommendations Assessment, Development and Evaluation (GRADE) [18]. It is divided into four grades: high, moderate, low, very low.

Ethics and dissemination

Ethical approval is not necessary because our study is not linked to individual patient data. In addition, the study findings will be published by peer reviewed journals.

Discussion

The incidence of IFP is not restricted by age and gender, and some special populations, such as pregnant women and diabetes patients, also have a higher incidence [19]. IFP also can increase the risk of cardiovascular disease, a research found that one in 60 people with the disease is at risk of death [20]. Furthermore, facial expression plays a significantly important role in interpersonal communications, and facial paralysis severely hinders this function [21]. Studies have shown that the poor recovery of facial muscle control and longer treatment cycles could brings

great mental torture and serious economic burden to the patients, and can even cause anxiety and depression, which seriously affects the work and daily life of patients [22, 23]. Therefore, there is an urgent need for an effective therapy to help patients relieve facial deformities and medical stress.

Acupuncture is one of the most unique treatments in the Chinese medicine system and has become a universal medicine advocated by the World Health Organization (WHO) and applied in many countries and regions. A findings in patients with facial paralysis revealed superiority of EA added to standard therapy over standard therapy alone in terms of improvement of nerve dysfunction, decrease in paralysis severity, and better functional recovery [24]. This indicate EA may be a safe and effective adjunct therapy for facial paralysis in achievement of more satisfactory clinical outcomes. Meanwhile, another study claimed EA is also suitable for the treatment of IFP [25]. However, there is no enough evidence to support these claims, the results of clinical studies on the efficacy and safety of EA in patients with IFP have been inconsistent too. This led us to the question: whether EA could be used to treat IFP and when and how it might be more effective. Therefore, this study aims to evaluate the efficacy and safety of EA for IFP patients by systematic review and meta-analysis, so as to provide clinical decision-making based on evidence-based medicine.

Supporting information

S1 Appendix. The PRISMA-P checklist.
(DOCX)

S2 Appendix. The search strategy for PubMed.
(DOCX)

S1 Fig. The PRISMA flow diagram of the study selection process.
(TIF)

Author Contributions

Conceptualization: Yihao Zhou, Jing Shi.

Data curation: Gan Huang, Chunhong Luo, Zhilin Huang.

Formal analysis: Yihao Zhou, Gan Huang.

Investigation: Jing Shi.

Methodology: Yihao Zhou, Anhong Dai.

Project administration: Meifang Liu, Jing Shi.

Resources: Gan Huang, Chunhong Luo, Zhilin Huang.

Software: Anhong Dai, Xuelian Zhang.

Supervision: Meifang Liu, Jing Shi.

Writing – original draft: Yihao Zhou.

Writing – review & editing: Yihao Zhou, Jing Shi.

References

1. Wu J. Neurology. Beijing: People's Medical Publishing House; 2010. 118–120.

2. Rath B, Gidudu JF, Anyoti H, et al. Facial nerve palsy including Bell's palsy: case definitions and guidelines for collection, analysis, and presentation of immunisation safety data. Vaccine. 2017; 35 (15):1972–1983. https://doi.org/10.1016/j.vaccine.2016.05.023 PMID: 27235092

3. Eviston TJ, Croxson GR, Kennedy PG, et al. Bell's palsy: aetiology, clinical features and multidisciplinary care. J Neurol Neurosurg Psychiatry. 2015; 86(12):1356–1361. https://doi.org/10.1136/jnnp-2014-309563 PMID: 25857657

4. Glass GE, Tzafetta K. Bell's palsy: a summary of current evidence and referral algorithm. Fam Pract. 2014; 31(6):631–642. https://doi.org/10.1093/fampra/cmu058 PMID: 25208543

5. Li L, Chen QQ. WEI Qing-lin's experience on intractable facial paralysis treated with acupuncture-moxibustion and massage. Zhongguo Zhen Jiu. 2013; 33(01):46–48.

6. Kleiss IJ, Hohman MH, Susarla SM, et al. Health-related quality of life in 794 patients with a peripheral facial palsy using the FaCE scale: a retrospective cohort study. Clin Otolaryngol. 2015; 40(6):651–656. https://doi.org/10.1111/coa.12434 PMID: 25858429

7. de Sire A, Marotta N, Agostini F, et al. A Telerehabilitation Approach to Chronic Facial Paralysis in the COVID-19 Pandemic Scenario: What Role for Electromyography Assessment?. J Pers Med. 2022; 12 (3):497. https://doi.org/10.3390/jpm12030497 PMID: 35330496

8. Shafshak TS. The treatment of facial palsy from the point of view of physical and rehabilitation medicine. Eura Medicophys. 2006; 42(1):41–47.

9. Teixeira LJ, Valbuza JS, Prado GF. Physical therapy for Bell's palsy (idiopathic facial paralysis). Cochrane Database Syst Rev. 2011;(12):CD006283. https://doi.org/10.1002/14651858.CD006283.pub3 PMID: 22161401

10. Wei YL, Liu J, Huang X, et al. Recent research on treating peripheral facial paralysis in TCM. Chinese Medical Journal. 2018; 10(13):135–138.

11. Li Y, Zhang X, Xie X, et al. Clinical research progress of electroacupuncture in the treatment of peripheral facial paralysis. Hunan Journal of Traditional. 2017; 33(07):186–188.

12. Kim SJ, Lee HY. Acute Peripheral Facial Palsy: Recent Guidelines and a Systematic Review of the Literature. J Korean Med Sci. 2020; 35(30):e245. https://doi.org/10.3346/jkms.2020.35.e245 PMID: 32743989

13. Guo F, Ruan CX, Ma Y, et al. Observation on the effect of combined acupuncture and medicine on the sequelae of peripheral facial paralysis and its influence on plasma endothelin. Zhejiang J Tradit Chin Med. 2010; 45(11):791–792.

14. Zhou X, Xiong J, Chi Z, et al. Effectiveness and safety of acupuncture and moxibustion for peripheral facial paralysis: A protocol for an overview of systematic reviews and meta-analysis. Medicine (Baltimore). 2020; 99(38):e22371. https://doi.org/10.1097/MD.0000000000022371 PMID: 32957415

15. Chen YH, Su XQ, Sun ZR, et al. Clinical observation on electroacupuncture with sparse-dense wave for idiopathic facial paralysis. Shanghai J Acupunct Moxi. 2013; 32(08):653–654.

16. Wang WH, Jiang RW, Liu NC. Electroacupuncture Is Effective for Peripheral Facial Paralysis: A Meta-Analysis. Evid Based Complement Alternat Med. 2020; 2020:5419407. https://doi.org/10.1155/2020/5419407 PMID: 32328134

17. Shamseer L, Moher D, Clarke M, et al. Preferred reporting items for systematic review and meta-analysis protocols (PRISMA-P) 2015: elaboration and explanation. BMJ. 2015; 350:g7647. https://doi.org/10.1136/bmj.g7647 PMID: 25555855

18. Balshem H, Helfand M, Schünemann HJ, et al. GRADE guidelines: 3. Rating the quality of evidence. J Clin Epidemiol. 2011; 64(4):401–406. https://doi.org/10.1016/j.jclinepi.2010.07.015 PMID: 21208779

19. Cao EM, Sun SB. Clinical Observation on Treatment of Intractable Facial Paralysis by Penetrating Needling Combined with Electro-acupuncture. Clinical journal of traditional chinese medicine. 2019; 31 (03):570–572.

20. Cave JA. Recent developments in Bell's palsy: does a more recent single research paper trump a systematic review?. BMJ. 2004; 329(7474):1103–1104. https://doi.org/10.1136/bmj.329.7474.1103-a PMID: 15528634

21. Razfar A, Lee MK, Massry GG, et al. Facial Paralysis Reconstruction. Otolaryngol Clin North Am. 2016; 49(2):459–473. https://doi.org/10.1016/j.otc.2015.12.002 PMID: 26902979

22. Lee SY, Kong IG, Oh DJ, et al. Increased risk of depression in Bell's palsy: Two longitudinal follow-up studies using a national sample cohort. J Affect Disord. 2019; 251:256–262. https://doi.org/10.1016/j.jad.2019.03.059 PMID: 30933749

23. Tseng CC, Hu LY, Liu ME, et al. Bidirectional association between Bell's palsy and anxiety disorders: A nationwide population-based retrospective cohort study. J Affect Disord. 2017; 215:269–273. https://doi.org/10.1016/j.jad.2017.03.051 PMID: 28359982

24. Gökçe Kütük S, Özkan Y, Topuz MF, et al. The Efficacy of Electro-Acupuncture Added to Standard Therapy in the Management of Bell Palsy. J Craniofac Surg. 2020; 31(7):1967–1970. https://doi.org/10.1097/SCS.0000000000006537 PMID: 32472874

25. Fabrin S, Soares N, Regalo SC, et al. The Effects of Acupuncture on Peripheral Facial Palsy Sequelae after 20 Years via Electromyography. J Acupunct Meridian Stud. 2015; 8(5):245–248. https://doi.org/10.1016/j.jams.2015.01.006 PMID: 26433801
